# Digital Interventions for Palliative Care Education for Nursing Students: A Systematic Review

**DOI:** 10.3390/nursrep16010016

**Published:** 2026-01-07

**Authors:** Abdulelah Alanazi, Gary Mitchell, Fadwa Naji Al Halaiqa, Fadi Khraim, Stephanie Craig

**Affiliations:** 1 School of Nursing and Midwifery, Queen’s University Belfast, Belfast BT7 1NN, UK; gary.mitchell@qub.ac.uk; 2 Department of Nursing, Faculty of Applied Medical Sciences, University of Bisha, Bisha 61922, Saudi Arabia; 3 Pre-Clinical Affairs, College of Nursing, Qatar University, Doha P.O. Box 2713, Qatar; f.alhalaiqa@qu.edu.qa (F.N.A.H.); fkhraim@qu.edu.qa (F.K.)

**Keywords:** palliative care, nursing education, digital learning, nursing students, digital, education, systematic review

## Abstract

**Background/Objectives**: Palliative care education is a core component of undergraduate nursing preparation; however, many nursing students report limited exposure and confidence in providing end-of-life care. Digital and web-based educational approaches have increasingly been adopted to address gaps in palliative care training and to provide flexible, scalable learning opportunities. This mixed-methods systematic review examined the use of digital and web-based approaches in palliative care education for pre-registration nursing students. The aim was to synthesize existing evidence on educational outcomes, confidence development, practice preparation, and acceptability to guide future design and implementation of technology-enhanced learning in this field. **Methods**: The review followed the Preferred Reporting Items for Systematic Reviews and Meta-Analyses (PRISMA) framework. The search was conducted across Medline (Ovid), Embase, CINAHL, Scopus and PsycINFO in October 2025. Studies employing qualitative, quantitative, or mixed-methods designs were eligible if they evaluated fully digital or web-based palliative care educational interventions for nursing students. Screening, quality appraisal, and data extraction were undertaken independently by multiple reviewers. Methodological quality was assessed using the Mixed Methods Appraisal Tool (MMAT). Extracted data were synthesized narratively to integrate qualitative and quantitative findings. **Results**: The search yielded 1826 records; after removing duplicates and applying eligibility criteria, 12 studies were included in the final synthesis. Considerable heterogeneity in design and outcomes was observed. Most included studies reported improvements in students’ knowledge, self-efficacy, and reflective capacity, alongside high levels of acceptability. **Conclusions**: Digital and technology-enhanced learning appears feasible and acceptable for palliative care education; however, the current evidence base is limited by methodological heterogeneity, reliance on self-reported outcomes, and predominantly short-term evaluations. Further rigorous, large-scale studies with objective outcome measures are required to determine sustained educational and practice impact.

## 1. Introduction

Palliative care is an essential component of holistic healthcare that seeks to improve the quality of life of patients and families facing life-limiting illness by addressing physical, psychological, social, and spiritual needs [1]. It is recognized internationally as a fundamental human right, supported by organizations such as the European Association for Palliative Care (EAPC), the International Association for Hospice and Palliative Care (IAHPC), and Human Rights Watch [1,2]. The World Health Organization (WHO) further emphasizes that palliative care should begin at the point of diagnosis and continue throughout the course of illness, not only at the end of life [3]. Providing this care requires healthcare professionals to be equipped with the knowledge, skills, and confidence for person-centered and ethically grounded practice from early illness through to bereavement.

The development of these competencies is particularly critical for nurses, who represent the future workforce and will provide much of the ongoing care in hospitals, care homes, hospices, and community settings [4]. Evidence indicates that structured and early exposure to palliative care education improves knowledge, attitudes, and emotional preparedness for end-of-life care [5]. Conversely, inadequate preparation is associated with moral distress, avoidance of dying patients, and increased risk of burnout [6]. Developing a sound foundation in palliative and end-of-life care during pre-registration education is therefore essential to ensure future nurses can deliver compassionate, competent, and person-centered care.

Although palliative care is inherently multidisciplinary, the educational context and professional roles of nursing students differ substantially from those of medical, pharmacy, or allied health students. Nursing education alternates frequently between theoretical academic study and practical clinical placements [7]. During placements, nursing students often work in settings, such as care homes or community environments, where they may serve as the primary point of contact for patients and families and act as conduits between the multidisciplinary team members (e.g., doctors, pharmacists, and therapists) [7,8]. This contrasts with medical and pharmacy students who typically learn within structured, hospital-based teams with readily available supervision. The nursing student’s experience is therefore distinct in both pedagogical and professional terms, demanding an education that prepares them to exercise autonomy, manage emotional complexity, and communicate effectively across professional and organizational boundaries. For this reason, this review focuses specifically on pre-registration nursing students. Evidence from other areas of nursing practice further highlights the importance of targeted educational interventions, with structured education shown to significantly enhance nurses’ clinical skills and decision-making capabilities across diverse care contexts [9].

The rapid expansion of digital interventions offers new opportunities to address these educational challenges. The COVID-19 pandemic accelerated the adoption of online teaching, simulation, and blended learning, demonstrating the potential of digital methods to deliver flexible, scalable, and interactive instruction [10,11]. Digital interventions such as web-based modules, virtual simulations, serious games, and digital storytelling can accommodate diverse learning preferences and allow students to revisit complex or emotionally demanding content at their own pace [12,13,14,15]. Digital interventions have been shown to enhance engagement, accessibility, and learning outcomes across health professions education [16]. Specifically, in palliative care, technology-enhanced education has been linked to improved confidence in communication, symptom management, and ethical decision-making [17]. For nursing students, digital learning provides continuity during placement rotations, supports independent study, and facilitates emotional safety in engaging with sensitive topics such as dying, loss, and grief.

Despite these advances, evidence on digital interventions for palliative care education in nursing students remains limited and fragmented, with variability in intervention types, outcome measures, and methodological rigor. Existing systematic reviews have often combined findings from multiple professional groups or focused narrowly on specific teaching techniques, making it difficult to draw definitive conclusions about the most effective strategies for undergraduate nursing education [4,5,10,14]. Furthermore, considerable heterogeneity exists in study design, intervention type, and outcome measures, which complicates synthesis and limits the transferability of findings [18]. There is therefore a clear need for an integrated analysis of how digital learning approaches are being used in nursing palliative care education, what outcomes they achieve, and how acceptable they are to students.

This review addresses that critical gap by systematically identifying, appraising, and synthesizing evidence from qualitative, quantitative, and mixed-methods studies evaluating digital or web-based palliative care education specifically for pre-registration nursing students. It aims to integrate data on learning outcomes and learner experiences to provide a comprehensive understanding of how digital education contributes to the development of palliative care competence. The findings are intended to inform educators, curriculum developers, and policymakers about effective and acceptable strategies for delivering palliative care education in nursing programs and to guide future research.

Accordingly, the aim of this systematic review was to identify and explore digital educational interventions used in palliative care education for pre-registration nursing students, to determine their effectiveness, acceptability, and potential impact on practice. The review was conducted in line with the Preferred Reporting Items for Systematic Reviews and Meta-Analyses (PRISMA) framework [19] to ensure methodological transparency and rigor.

## 2. Materials and Methods

### 2.1. Review Protocol

This review was conducted and reported in accordance with the Preferred Reporting Items for Systematic Reviews and Meta-Analyses (PRISMA) guidelines to ensure transparency in reporting [19] (Supplementary File S1). This review adopted an integrated approach, synthesizing both qualitative and quantitative research to thoroughly explore Digital Interventions in Palliative Care Education for Nursing Students. The review protocol detailed the search strategy, eligibility criteria, screening processes, quality appraisal, data extraction, and synthesis procedures. The protocol was developed prospectively prior to data extraction and registered on the Open Science Framework (OSF) to enhance transparency and replicability (https://osf.io/ukmva/overview accessed on 26 October 2025). No deviations from the protocol occurred.

### 2.2. Search Strategy

A comprehensive search strategy was developed in consultation with an academic librarian, following systematic review search guidance by Aromataris and Riitano [20]. Five databases were selected for their relevance to nursing and healthcare education: MEDLINE (Ovid), Embase, CINAHL, Scopus and PsycINFO (Table 1). The full search strategy used in CINAHL can be accessed in Supplementary File S2.

The database searches were conducted in October 2025. Keywords were derived from three core concepts: digital education, nursing students, and palliative care. Controlled vocabulary (MeSH terms) and Boolean operators were applied to ensure a comprehensive and replicable search strategy, incorporating synonyms and related terms such as online learning, virtual simulation, serious games, and end-of-life care (Table 2). No date or publication limits were applied to maximize retrieval of relevant evidence.

### 2.3. Inclusion and Exclusion Criteria

Eligibility criteria were developed a priori to minimize selection bias and ensure consistency during screening across population, intervention, outcomes, and study-design domains [21]. Studies were eligible for inclusion if they involved pre-registration or undergraduate nursing students and evaluated digital or web-based educational interventions delivered as part of a university program. Interventions were required to focus on palliative or end-of-life care, or to include a distinct palliative care component. All qualitative, quantitative, and mixed-methods research designs were eligible, including peer-reviewed journal articles, conference abstracts, and dissertations, provided that data relating to palliative care learning outcomes were reported. Gray literature sources were searched; however, no eligible studies met the inclusion criteria.

Studies were excluded if they targeted non-nursing participants or were designed primarily for other professional groups, employed in-person or non-digital teaching methods, focused on post-registration or hospital-based training, or represented non-empirical publications such as commentaries, guidelines, or opinion pieces.

Digital interventions were defined as fully online or computer-mediated educational activities including; web-based modules, virtual simulation, digital storytelling, serious games, interactive case-based scenarios, and VR-based experiences. Blended or partially face-to-face interventions were excluded unless the digital component could be evaluated independently, ensuring that effects were attributable to digital learning.

### 2.4. Screening and Study Selection

All retrieved records were imported into Covidence (https://www.covidence.org/ accessed on 20 December 2025) a reference management software for automatic duplication and to support data management and extraction. Title and abstract screening were conducted independently by two reviewers (AA and GM) against the predefined eligibility criteria, with a third reviewer (SC) available for consultation where uncertainty arose. Full-text screening was undertaken independently by AA, GM, any discrepancies were resolved through discussion and consensus, with arbitration by (SC) when required [22,23].

### 2.5. Data Extraction

Data extraction followed established guidance [24,25]. A structured form, piloted on a subset of studies, was used to capture key information including author, year, aim, study design and methods, intervention format, educational context, population and sample characteristics, and outcomes related to digital palliative care education. Two reviewers (A.A. and G.M.) independently extracted data and cross-checked entries for accuracy, with discrepancies resolved through discussion and, where necessary, consultation with a third reviewer (S.C.).

### 2.6. Quality Appraisal

All included studies were assessed for methodological quality using the Mixed Methods Appraisal Tool (MMAT, 2018) [26], which allows appraisal across qualitative, quantitative, and mixed-methods designs. Quality assessment was conducted independently by three reviewers (A.A., G.M., and S.C.), with discrepancies resolved through discussion until consensus was achieved. No studies were excluded based on methodological quality, as all met the minimum acceptable standards for inclusion. Seven studies were rated as high quality, typically mixed-methods or well-structured experimental designs while five studies were rated as moderate quality owing to smaller sample sizes or limited methodological controls such as lack of blinding (Supplementary File S3). This approach ensured that the synthesis was grounded in evidence of adequate methodological robustness while remaining inclusive of emerging study designs such as quasi-experimental and virtual reality–based interventions.

While no studies were excluded on the basis of quality, methodological ratings were used to inform interpretation of findings during synthesis. Greater analytical weight was given to higher-quality studies, and findings from moderate-quality studies were interpreted cautiously to avoid overstatement of effect.

### 2.7. Data Synthesis

Given the substantial heterogeneity in intervention formats, study designs, and outcome measures, a narrative synthesis approach was adopted as the most appropriate method for integrating quantitative and qualitative evidence within this mixed-methods systematic review. Narrative synthesis is recommended when statistical meta-analysis is not feasible and when the aim is to explore patterns, mechanisms, and contextual influences across diverse study types [27]. This approach aligns with JBI and MMAT guidance for mixed-methods reviews, allowing both numerical and textual data to be systematically interpreted to address complex educational outcomes [24,26,28].

The synthesis followed the framework outlined by Popay [29], incorporating four stages: (1) developing a preliminary synthesis, (2) exploring relationships within and between studies, (3) assessing the robustness of the evidence, and (4) drawing integrated conclusions. Extracted data were tabulated and coded line by line to identify recurrent concepts and outcome patterns. Findings were subsequently grouped into four over-arching analytical themes: knowledge, self-efficacy/confidence, practice, and acceptability/implementation. Coding and thematic refinement were conducted collaboratively by AA, GM, and SC, with supervisory oversight and consensus achieved through iterative discussion. Integration of qualitative and quantitative evidence is illustrated through a joint display (Table 3), which maps convergent findings across analytical themes and demonstrates how diverse data sources collectively inform conclusions regarding knowledge, confidence, practice, and acceptability.

### 2.8. Characteristics of Interventions and Participants

To provide a structured synthesis, all eligible studies were examined to determine the nature of the digital interventions and the characteristics of participating. Interventions were reviewed and categorized based on primary instructional design and delivery format (e.g., web-based modules, simulations, digital storytelling). Four broad categories were identified during data extraction: web-based modules [4,31,33,37], virtual simulations [32,34,35,36,38], digital storytelling activities [8], and online courses incorporating video lectures and discussion forums [18,30]. This categorization enabled consistent comparison of pedagogical approaches across studies. For each included study, information on participant characteristics was systematically extracted, including sample size, academic level, age, gender distribution, and, where available, ethnicity. When mixed cohorts (e.g., nursing, medical, or allied health students) were reported, data relating specifically to nursing students were isolated for analysis. This approach ensured that the synthesis focused exclusively on the nursing student population targeted in this review.

## 3. Results

### 3.1. Study Characteristics

**Figure 1:**
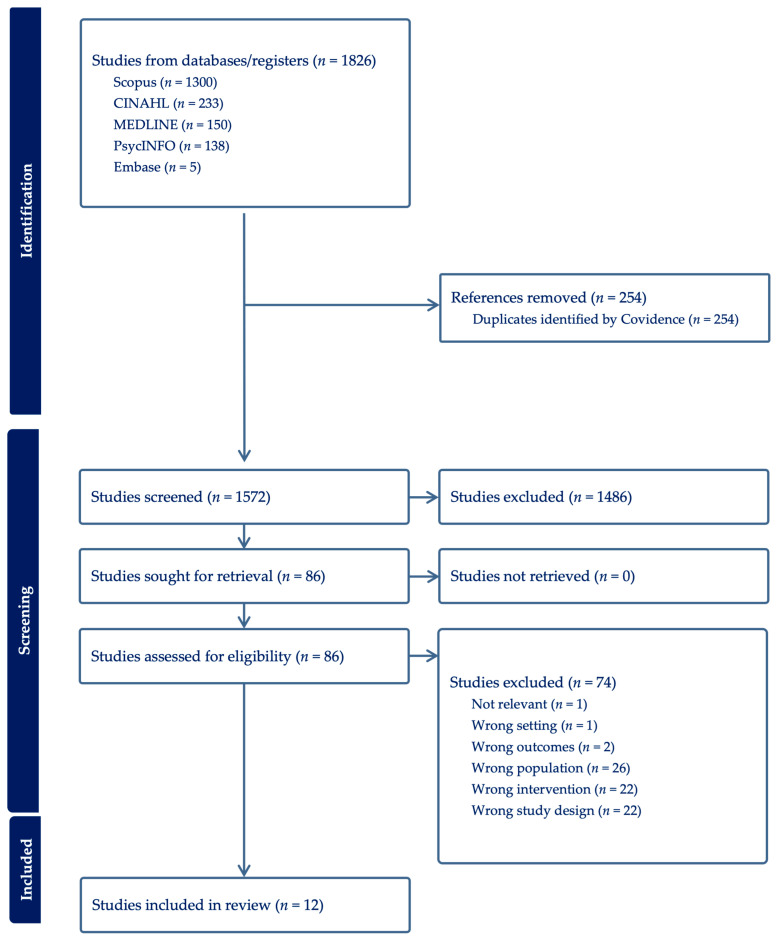
Preferred Reporting Items for Systematic Reviews and Meta-Analyses (PRISMA).

The search identified 1826 records; after removal of 254 duplicates, 1572 records were screened by title and abstract. Eighty-six full-text articles were assessed for eligibility, of which 74 were excluded. Twelve studies met the inclusion criteria and were included in the final synthesis. Records and screening were managed using Covidence. A total of 12 studies published between 2012–2024 were included in this review. The study selection process is presented in the PRISMA flow diagram (Figure 1).

The studies were conducted across the United States (*n* = 7), Turkey (*n* = 2), South Korea (*n* = 1), Canada (*n* = 1) and China (*n* = 1). Most study samples involved undergraduate nursing students, the combined sample across all included studies was approximately 13,741 participants; however, this figure was disproportionately driven by a single large-scale study that primarily reported student satisfaction and perceived usefulness outcomes, while most other included studies involved substantially smaller samples and more detailed educational and clinical outcome measures. The digital interventions evaluated across these studies included web-based modules, virtual simulations, digital storytelling, and serious games. Key methodological characteristics, participant demographics, and intervention descriptions are summarized in Table 4.

### 3.2. Study Results

Although most included studies reported positive educational outcomes, the overall strength of evidence was limited by heterogeneity in study design, small sample sizes, and heavy reliance on self-reported measures. These limitations were taken into account when interpreting thematic findings.

Across the included studies, four overarching themes were identified regarding the effects of digital palliative-care education on nursing students: (1) Knowledge; (2) Confidence; (3) Practice; and (4) Acceptability and Implementation.

#### 3.2.1. Theme One: Knowledge

Digital education has emerged as a significant pedagogical approach in palliative care training, offering flexible, interactive, and student-centered methods to develop theoretical understanding and clinical reasoning. Across the included studies, digital platforms consistently improved nursing students’ knowledge and comprehension of palliative care concepts such as symptom control, communication, ethics, and psychosocial support. Conner, Price, and Mazanec [8,30,31] reported that online learning modules significantly improved students’ knowledge toward end-of-life care, reflecting how structured digital learning can foster both cognitive and affective gains. The findings suggest that online platforms provide a psychologically safe environment for exploring emotionally charged topics that may be avoided in traditional classroom discussions. Similarly, Akdeniz Kudubes and Bektas [4] noted that the web-based pediatric palliative-care course marked increases in knowledge and comprehension, supported by interactive case scenarios, self-tests, and multimedia content that encouraged repeated engagement and reflection.

These studies indicate that interactivity, psychological safety, flexibility, and self-paced repetition are key mechanisms underpinning the effectiveness of digital learning. Shrestha [33] observed that a 90 min asynchronous e-learning module on end-of-life decision-making significantly increased both perceived knowledge and confidence, with learners highlighting the value of multimedia case narratives and reflective questions in linking theory to practical decision-making frameworks. Similarly, Mazanec [31] found that the End-of-Life Nursing Education Consortium (ELNEC) online curriculum improved understanding of pain management, ethics, and communication, demonstrating that structured combinations of text, video, and reflection enhance knowledge retention. Digital storytelling also emerged as a particularly powerful approach, with Price [8] reporting that narrative-based learning strengthened emotional engagement and cognitive recall by connecting abstract theory with authentic patient experiences. These findings are consistent with experiential and constructivist learning theories, suggesting that emotionally anchored content supports the internalization of professional values and patient-centered care principles. Zhang [32] further noted that simulation-based learning promoted deeper cognitive engagement, although increasing the realism of virtual patients may strengthen transfer to clinical practice. Sample sizes varied significantly across studies, ranging from small pilot cohorts to large national cohorts, which affects the weight of evidence.

Moreover, digital education mitigates barriers of geography and scheduling, broadening access to specialized palliative-care content. While the studies vary in scope and design, the convergence of evidence supports the potential effectiveness of online modules, simulations, and storytelling initiatives in improving nursing students’ understanding of palliative-care principles and perceived preparedness for clinical application. Future research should now focus on enhancing simulation realism and evaluating the durability of these knowledge gains in clinical settings.

#### 3.2.2. Theme Two: Confidence

Confidence and self-efficacy are critical determinants of clinical competence, especially in palliative care, where psychological readiness and emotional resilience directly influence the quality of patient support [39]. Through the included studies, digital educational interventions consistently enhanced students’ confidence in providing palliative and end-of-life care by offering safe, iterative, and emotionally supportive learning environments. Kasar [18] noted that nursing students who participated in an online course on ethical and clinical aspects of palliative care showed a significant increase in self-efficacy (*p* < 0.05). Similarly, Zhang [32] found that virtual clinical simulations enabled students to repeatedly practice complex decision-making scenarios, resulting in measurable gains in confidence and preparedness for real clinical encounters. The repetitive, feedback-driven structure of these simulations allowed students to refine their skills incrementally, reinforcing the role of deliberate practice in confidence building.

These findings are further supported by Jeon [35], whose study on addressing challenging communication situations for nursing students, revealed substantial improvements in students’ confidence when engaging with grieving caregivers and coordinating post-death procedures. Although only one scenario explicitly addressed end-of-life care, participants reported feeling better equipped to manage emotionally intense conversations and provide compassionate reassurance to families. Qualitative reflections from this study such as students noting, “My confidence grew with each simulation session” highlight the importance of experiential repetition in reducing anxiety and promoting a sense of clinical readiness. Lewis [34] also found that combining web-based learning with virtual simulations significantly strengthened students’ self-reported readiness to provide palliative care, underscoring the importance of multi-modal learning environments that blend theoretical knowledge with applied practice.

Immersive technologies have emerged as particularly effective tools for strengthening emotional resilience and confidence. Flood [36] demonstrated that virtual reality (VR) experiences where students embodied the perspective of a terminally ill patient, produced notable increases in self-efficacy across all stages of end-of-life care, from symptom management to final moments of life, and some students reported emotional fatigue and cybersickness during the VR scenarios, indicating that psychological and physical safety measures are important during immersive intervention. Importantly, confidence gains were accompanied by reductions in fear and helplessness, suggesting that controlled exposure to emotionally intense experiences within a virtual environment can reduce anxiety and enhance psychological preparedness.

However, while digital interventions generally promote confidence, several challenges remain. Zhang [32] cautioned that insufficient realism in some simulations may limit emotional immersion, thereby weakening the authenticity of confidence-building exercises. Additionally, the diversity in students’ technological proficiency and emotional coping styles means that confidence gains are not uniformly distributed. Flood [36] also mentioned that immersive experiences, though powerful, can occasionally induce emotional fatigue or cybersickness, necessitating structured debriefing sessions to ensure psychological safety. These nuances highlight the importance of tailoring confidence-building interventions to individual needs and ensuring robust pedagogical and emotional support.

Overall, the collective evidence demonstrates that digital education significantly enhances nursing students’ confidence in delivering palliative care by combining repetition, feedback, and safe emotional exposure. These interventions bridge the gap between theoretical learning and the affective demands of real-world practice, enabling students to approach end-of-life care with greater assurance, empathy, and composure. Consequently, digital learning environments not only cultivate technical competence but also nurture the psychological resilience essential for compassionate palliative practice.

#### 3.2.3. Theme Three: Practice

The translation of theoretical knowledge into competent clinical practice represents one of the most important goals of palliative care education. Digital educational interventions, particularly those incorporating interactivity and realism, have proven highly effective in bridging the gap between academic learning and real-world application. Across reviewed studies, virtual simulations, web-based modules, and narrative-based learning consistently enhanced students’ communication, decision-making, and clinical reasoning skills, preparing them to deliver compassionate, evidence-based palliative care.

A key example of this translation is demonstrated in Wittenberg’s study [37], where the COMFORT™ SM communication module improved students’ ability to manage sensitive conversations with dying patients and their families. The structured framework encouraged students to practice empathy, active listening, and emotional regulation skills central to high-quality palliative care. Jeon [35] found that technology-based simulations of end-of-life caregiver interactions enhanced preparedness and emotional composure. The study facilitated the transfer of communication strategies from simulated exercise to other emotionally charged clinical contexts, reflecting a broader application of learned competencies. Together, these findings highlight how digital interventions can create psychologically safe environments for practicing emotionally demanding interactions, helping students develop communication proficiency before entering clinical placements.

Virtual simulations also played a critical role in improving clinical decision-making and holistic assessment skills. Zhang [32] reported that repeated participation in immersive simulations allowed students to integrate biomedical knowledge with psychosocial and ethical considerations when managing complex cases. This iterative process fostered the development of clinical reasoning, situational awareness, and reflective judgment. Similarly, Ellman [38] demonstrated that simulation-based learning supported students in applying theoretical principles to practice, leading to improved confidence and adaptability in real-world palliative contexts. These studies suggest that simulation functions as a “translational space,” enabling students to bridge cognitive understanding and behavioral application in a low-risk, feedback-rich environment [40].

Beyond simulations, web-based programs and storytelling formats also enhanced practical competencies. Akdeniz and Bektas [4] found that structured online modules significantly improved students’ abilities in pain management, symptom control, and end-of-life communication (all *p* < 0.05). The inclusion of interactive exercises and clinical scenarios encouraged active engagement and decision-making, effectively simulating real-world problem-solving. Complementarily, Price [8] also illustrated that digital storytelling deepened students’ empathy and understanding of patient experiences, promoting reflective practice and a more humanistic approach to care. Through constructing and reflecting on patient narratives, students were able to internalize ethical and emotional dimensions of clinical work that are often underemphasized in traditional teaching.

Structured digital curricula such as the ELNEC online modules [31] further reinforced this pattern by combining repetition, reflection, and evidence-based frameworks. Students reported not only increased practical competence but also greater confidence in addressing ethical dilemmas and communication challenges. Flood [36] also noted that immersive virtual reality experiences where students embodied a dying patient prompted powerful behavioral intentions to modify future practice, including improved communication, dignified body handling, and advocacy for patient autonomy. Such immersive methods extend learning beyond skill acquisition toward deeper attitudinal and ethical transformation [41]. Although practice improvements were consistently reported, their magnitude differed by intervention type and assessment method.

These studies confirm that digital interventions promote the integration of theoretical, emotional, and ethical learning into practical competence. By providing opportunities for repetition, reflection, and emotional engagement, digital modalities cultivate clinical reasoning, communication proficiency, and compassionate professionalism skills essential for effective palliative care. Although issues such as simulation realism and technological variability persist, the evidence strongly supports digital education as an innovative and effective strategy for improving both the technical and moral dimensions of nursing practice in palliative contexts.

#### 3.2.4. Theme Four: Acceptability and Implementation

While technological innovation has enhanced flexibility, accessibility, and engagement, its implementation also presents structural, emotional, and pedagogical challenges. Across the studies reviewed, facilitators were associated with learner engagement, curricular adaptability, and institutional support, whereas barriers primarily involved technological limitations, emotional strain, and variability in faculty readiness. Among the most prominent facilitators, engaging pedagogical strategies such as digital storytelling, virtual simulations, and interactive case-based learning emerged as crucial to student acceptability. Price [8] identified digital storytelling as a particularly effective medium for fostering creativity and emotional engagement. Through narrative-based learning, students were able to humanize complex palliative care concepts, internalize patient perspectives, and cultivate empathy. The reflective and imaginative nature of storytelling enhanced both understanding and memory retention, illustrating how emotional resonance can strengthen learning outcomes. Mazanec [31] emphasized the importance of curricular flexibility in digital platforms, noting that online formats allowed students to access materials at their convenience and adapt learning to their pace and style. This flexibility not only improved satisfaction and accessibility but also contributed to the scalability of digital interventions across diverse nursing programs. The psychological safety of virtual simulations was another key facilitator. Jeon [35] reported that students valued the ability to rehearse emotionally charged end-of-life conversations within a risk-free environment. The opportunity to make mistakes and receive feedback without real-world consequences promoted both confidence and skill mastery.

Despite these strengths, several barriers to implementation persist. Technological and design-related limitations were among the most frequently cited. Zhang [32] and Flood [36] observed that some virtual simulations lacked realism, which reduced emotional immersion and hindered transferability of learning to clinical contexts. Enhancing the authenticity of virtual patient interactions was identified as a critical improvement area to ensure fidelity between simulation and real practice. The studies also noted that while immersive VR simulations elicited deep empathy and engagement, some students experienced cybersickness or emotional overwhelm. The absence of structured debriefing limited reflective integration, underscoring the need for post-simulation support to ensure psychological safety in emotionally intense learning environments.

Structural and interpersonal barriers were also evident; Conner [30] and Ellman [38] highlighted challenges in interprofessional digital programs, where unequal representation of different healthcare disciplines hindered collaboration and shared learning. Balanced participation across professional groups is vital for achieving holistic palliative care perspectives. Moreover, the studies found that peer-assessment components, while pedagogically valuable, sometimes created discomfort among students reluctant to critique peers’ work. These findings highlight the importance of designing supportive peer-learning structures that foster collaboration rather than competition.

Faculty-related barriers further complicated implementation, Mazanec [31] noted that perceived costs, time investment, and lack of training in digital pedagogy limited integration into curricula. Institutional support, including professional development and technological infrastructure, is therefore essential for sustaining program quality and faculty engagement.

Overall, the literature demonstrates that the acceptability and effectiveness of digital palliative care education hinge on balancing innovation with empathy, realism, and institutional readiness. Facilitators such as narrative-based pedagogy, flexible learning design, and safe simulation environments enhance engagement and accessibility. However, addressing barriers particularly technological limitations, emotional strain, structural imbalances, and insufficient faculty preparation is crucial for maximizing the potential of digital education. Through strategic investment in technology, faculty training, and psychological support mechanisms, educational institutions may enhance the conditions under which digital palliative care education can be implemented effectively and sustainably.

## 4. Discussion

This discussion synthesizes findings from twelve studies examining how digital educational interventions influence pre-registration nursing students’ knowledge, confidence, and clinical practice in palliative care [4,8,18,30,31,32,33,34,35,36,37,38]. The evidence confirms that well-designed digital modalities enhance both cognitive and affective learning outcomes, aligning with constructivist and connectivism theories which emphasize learner-centered engagement, reflection, and iterative feedback [28,32,42]. The results are encouraging but showcase the necessity for ongoing refinement to ensure these gains translate into sustainable professional competence.

Knowledge improvement was the most frequently reported outcome. Web-based programs, such as the pediatric palliative care course [4] and the ELNEC online curriculum [31], significantly enhanced students’ understanding of pain management, communication, and ethics. This aligns with broader nursing-education findings that interactive digital tools enhance comprehension and retention [33]. However, none of the included studies assessed long-term retention or performance, limiting conclusions about sustained competence. Consistent with broader nursing education research [43,44], the use of interactive and repeatable learning fosters retention and deeper conceptual understanding. However, a critical gap remains, as most studies assessed only short-term knowledge gains; the long-term impact on professional practice is insufficiently explored [33].

The strength of the conclusions drawn in this review is constrained by the methodological limitations of the included evidence. Most studies employed quasi-experimental or single-group pre–post designs and relied primarily on self-reported measures of knowledge, confidence, and attitudes. Few studies incorporated objective assessments of clinical performance, behavioral change, or long-term follow-up. These limitations reduce certainty regarding the transfer of learning into sustained clinical competence.

Digital education also effectively strengthened students’ self-efficacy and confidence. Simulation-based and interactive learning enhanced preparedness for emotionally complex situations [18,32,35,36]. These outcomes reflect Bandura’s theory [45] that mastery experiences and feedback build self-belief. By allowing safe, repeated practice, digital tools reduce anxiety and reinforce confidence in clinical decision-making. Specifically, Flood [36] noted that students who virtually embodied a dying patient reported higher confidence and reduced fear, suggesting that emotionally immersive experiences can foster both competence and empathy. Nevertheless, the literature highlights that increased self-reported confidence does not always correlate with practical proficiency; mentorship and supervised clinical exposure remain necessary to consolidate skills [38]. Across studies, outcome measures varied substantially, ranging from self-efficacy scales to communication-performance rubrics. This heterogeneity limits comparability and weakens the ability to determine which digital modalities are most effective.

Practice-related outcomes were notable across various digital modalities. Virtual simulations strengthened decision-making and end-of-life communication skills [32,35]. Wittenberg [37] found that a communication module improved students’ ability to manage difficult conversations, while Akdeniz and Mazanec [4,31] highlighted improvements in pain control and ethical reasoning. These studies consistently noted that simulations provided safe, reflective environments for practicing complex interactions, receiving feedback, and building competence in managing sensitive palliative care scenarios [32,35]. Furthermore, digital storytelling deepened reflection and empathy [8], with immersive VR experiences influencing knowledge, confidence, and prompting behavioral intentions to modify communication and advocacy practices [36]. These findings confirm that digital learning can effectively bridge theoretical knowledge and applied competence, although integration with hands-on training remains essential.

A key pedagogical mechanism underpinning many of the observed learning gains is the role of affective learning [46], whereby emotionally anchored content facilitates the transformation of knowledge into compassionate practice. Narrative-based approaches, such as digital storytelling, and immersive modalities, including virtual reality, engage learners at both emotional and cognitive levels, supporting reflection, empathy, and professional identity formation. Studies employing digital storytelling and embodied or simulated patient experiences demonstrated that emotional engagement enhanced students’ ability to internalize palliative care values, contextualize clinical decision-making, and apply knowledge with sensitivity and compassion in practice [8,36]. In contrast, purely cognitive gains achieved through information-based digital modules may improve factual knowledge but are less likely to foster the emotional readiness required for end-of-life care. This synthesis aligns with experiential learning theory and reflective practice models, which emphasize that learning is deepened when experience, emotion, and reflection are integrated to support meaning-making and behavioral change [42,47].

Student acceptance of digital education was overwhelmingly positive. Flexibility and accessibility were primary advantages, allowing students to manage learning around clinical commitments [31,33]. Storytelling increased engagement and emotional connection [8], and simulations were perceived as highly useful when accompanied by clear instruction and structured debriefing [32,35]. However, barriers persist [48]. Limited simulation realism [32], reluctance toward peer assessment [34], and variable digital literacy [31] can hinder optimal engagement. Flood [36] also cautioned that immersive VR, though impactful, occasionally caused cybersickness or emotional overwhelm, underscoring the necessity for psychological safety and post-session support. Overall, digital education is an effective, acceptable, and increasingly essential component of palliative care training. It offers scalable, flexible, and interactive approaches suited to the evolving demands of healthcare education. Yet, the current variability in program quality and outcome measurement shows the importance of theory-driven design and robust long-term evaluation.

One included study, Ellman et al. [38], examined an interprofessional digital education intervention but identified unequal representation across professional groups as a structural limitation. This finding is particularly relevant in the context of palliative care, where effective practice depends on shared understanding, role clarity, and collaboration across multidisciplinary teams. Nurses often act as consistent points of contact and coordinators of care, translating multidisciplinary plans into day-to-day practice; consequently, imbalanced interprofessional participation in educational interventions may limit opportunities to develop collaborative competencies. Future digital palliative care education should therefore prioritize equitable interprofessional representation to support holistic and integrated care delivery.

One important consideration in interpreting these findings is the influence of a single large-scale study [31] with a substantially larger sample size than the remaining included studies. While this study contributed valuable evidence regarding the acceptability, feasibility, and scalability of digital palliative care education, its outcomes were largely limited to self-reported satisfaction and perceived usefulness. As such, it did not disproportionately inform conclusions related to knowledge acquisition, confidence development, or practice-related behaviors, which were primarily derived from smaller experimental and mixed-methods studies with more detailed educational outcome measures. To mitigate potential weighting bias, findings were interpreted thematically rather than quantitatively, and greater analytical emphasis was placed on methodological rigor and outcome relevance rather than sample size alone.

While the included studies identified faculty-related barriers such as time investment, cost, and training demands, the sustainability of digital palliative care education also warrants consideration of faculty workload and burnout. Technology-enhanced teaching frequently requires substantial preparatory time, ongoing management of technical challenges, and the facilitation of emotionally intensive end-of-life content, which may compound existing academic workload pressures. Evidence from nursing education indicates that high workload demands and limited psychological resources are significant contributors to job burnout among nursing faculty, with implications for teaching quality, staff retention, and the long-term viability of educational innovation [49]. Addressing faculty wellbeing through institutional support, protected teaching time, and training in digital pedagogy may therefore be essential for sustaining digital palliative care education. Overall, the findings of this review are best interpreted as proof-of-concept evidence for the potential of digital modalities in palliative care education rather than definitive or generalizable evidence for specific programs. Although consistent improvements in knowledge, confidence, and communication were observed, the predominance of single-institution, non-randomized studies limits broader inference. These findings therefore support cautious adoption alongside further large-scale, methodologically robust research.

This systematic review provided valuable insight into the immediate educational impact of digital palliative care interventions through its focus on short-term learning outcomes, consistent use of validated tools, and inclusion of a diverse range of intervention types [19,50]. However, several limitations must be acknowledged. The absence of long-term follow-up across included studies limits conclusions about the sustainability of knowledge acquisition and behavioral change. Furthermore, reliance on self-reported outcome measures introduces the potential for response bias, as reflected in recent evaluations of digital pain and palliative care education [50]. To advance the evidence base, future research should incorporate objective, performance-based outcome measures, such as structured clinical examinations, rater-evaluated simulation performance, or observed communication assessments, rather than relying solely on self-reported outcomes. In addition, longitudinal study designs with follow-up periods of at least six months are needed to determine whether learning gains are sustained and translate into behavioral change in clinical practice. In addition, the lack of cost-effectiveness analyses and limited consideration of technological disparities and digital literacy among participants must be acknowledged [31,32]. Equity of access also warrants attention, as students with limited internet access, outdated devices, or lower levels of digital competence may experience reduced benefit from these interventions. Furthermore, reliance on self-reported outcome measures introduces the potential for response bias, highlighting the need for future research to incorporate objective, performance-based assessments and longer-term follow-up.

Future research should assess the sustainability of learning, effective integration with clinical placements, and equitable access for learners with differing digital competencies. When aligned with sound pedagogy and adequate institutional support, digital education has the potential to prepare nurses who are not only knowledgeable and skilled but also emotionally equipped to deliver compassionate, patient-centered palliative care. These findings collectively illustrate that while digital modalities offer substantial pedagogical value, their effectiveness is contingent upon thoughtful design, pedagogical grounding, and integration with clinical practice opportunities.

## 5. Conclusions

This systematic review suggests that digital interventions represent a promising and increasingly important component of palliative care education for pre-registration nursing students. Current evidence suggests potential benefits for knowledge acquisition, confidence development, and perceived preparedness for practice. However, the evidence base remains limited by heterogeneity, reliance on self-reported outcomes, and a lack of long-term objective evaluation. Future research using more rigorous designs and comprehensive outcome measures is required before definitive conclusions regarding educational effectiveness can be drawn.

## Figures and Tables

**Table 1:** Description of databases and journals used in search.

Database	Description
MEDLINE (Ovid)	A comprehensive source of life sciences and biomedical bibliographic information. It covers a wide range of topics, including nursing, with extensive coverage of medical, clinical, and healthcare-related research. Articles from 1946 to present.
Embase	A biomedical and pharmacological database. It offers extensive coverage of drug and pharmaceutical research, as well as biomedical literature, making it highly relevant for nursing and healthcare topics. Articles from 1974 to present.
Cumulative Index to Nursing and Allied Health Literature (CINAHL)	Provides indexing for journals from the fields of nursing and allied health. It is a key resource for nursing professionals, offering extensive literature on patient care, nursing practices, and healthcare management. Articles from 1937 to present.
PsycINFO	A database of abstracts and citations in the field of psychology. It includes literature on psychological aspects of healthcare and nursing, making it a valuable resource for understanding the psychological elements of patient care. Articles from 1806 to present.

**Table 2:** Search Strategy.

Key Terms	MeSH Terms
Digital or web-based	Digital technology OR internet-based intervention OR Digital Learning OR E-Learning OR Online Learning OR Web-Based Learning OR Online Education OR Remote Teaching OR Remote Learning OR Mobile Learning OR Virtual Learning OR Serious Game OR serious games OR serious gam * OR Gamification OR Podcast OR Web-Based App OR Web-Based Application OR Web-Based Applications OR App OR MOOC
	AND
Nursing students Education	Nursing Education OR nursing Educational OR Nur * Education OR Nur * Educational OR Nursing OR Nurse OR Nurses Nursing Student OR students nursing OR Nur * stud * OR stud * Nur * OR Baccalaureate Nursing OR Bachelor nursing OR Undergraduate nursing OR pre-registration Nursing OR Baccalaureate Nur * OR Bachelor Nur * OR Undergraduate Nur * OR pre-registration Nur * OR student Nurse OR Stud * Nur * OR Nurses OR Nursing OR Nur *
	AND
Palliative care	Palliative care OR End-of-life care OR Hospice care OR EoLC OR PC OR Comfort care OR Supportive care OR Palliation OR advance care planning OR palliative OR symptom management OR end of life OR end of life decision making

* Truncation symbol used to retrieve multiple word variants from a common root.

**Table 3:** Integrated mixed-methods synthesis of qualitative and quantitative findings.

Theme	Quantitative Findings	Qualitative Findings	Included Studies
Knowledge	Significant post-intervention improvements in palliative care knowledge, including symptom management, communication, ethics, and end-of-life decision-making (*p* < 0.05 to *p* < 0.001 across studies).	Students reported deeper understanding of palliative care concepts, improved ability to link theory to practice, and greater awareness of holistic and person-centered care. Digital storytelling and simulation were perceived as particularly effective for conceptual learning.	Akdeniz & Bektas [4]; Conner et al. [30]; Mazanec et al. [31]; Zhang et al. [32]; Shrestha et al. [33]; Price et al. [8]
Confidence/Self-efficacy	Statistically significant increases in self-efficacy, confidence in communication, and preparedness for end-of-life care following online courses, simulations, and VR interventions.	Students described reduced fear, increased emotional readiness, and growing confidence when engaging with dying patients and families. Repeated practice and emotionally immersive experiences were viewed as central to confidence building.	Kasar [18]; Lewis-Pierre et al. [34]; Zhang et al. [32]; Jeon et al. [35]; Flood [36]
Practice	Improvements in self-reported palliative care practices, communication performance scores, and decision-making abilities following digital and simulation-based interventions.	Qualitative data highlighted enhanced empathy, reflective practice, ethical sensitivity, and perceived readiness to translate learning into clinical practice. Simulations functioned as a safe space to rehearse complex behaviors.	Akdeniz & Bektas [4]; Wittenberg et al. [37]; Ellman et al. [38]; Zhang et al. [32]; Jeon et al. [35]; Flood [36]
Acceptability and Implementation	High levels of satisfaction, perceived usefulness, and acceptability of digital palliative care education across diverse formats (modules, simulations, VR).	Students valued flexibility, psychological safety, and engagement. Barriers included technological limitations, limited realism, emotional fatigue, and need for structured debriefing and faculty support.	Mazanec et al. [31]; Price et al. [8]; Shrestha et al. [33]; Zhang et al. [32]; Flood [36]; Ellman et al. [38]

**Table 4:** Study Characteristics.

Author	Year	Country	Study Aim	Study Design	Sample	Intervention	Data Collection/Analysis	Key Findings	Limitations
Akdeniz and Bektas [4]	2020	Turkey	To investigate the effect of web-based pediatric palliative care education on nursing students’ knowledge level and practices related to palliative care.	Quantitative quasi-experimental study	Third-year nursing students enrolled in a pediatric nursing course, with 265 participants (133 control; 132 intervention).	A web-based pediatric palliative care education program consisting of eight modules.	Nursing Student Information Form: collected demographic data and baseline knowledge. Palliative Care Knowledge Level Form: measured knowledge about pediatric palliative care. Scale of Self-Reported Palliative Care Practices (SRPCP): evaluated self-reported practices in palliative care. Data analysis: mean and percentage calculations, *t*-tests, regression analysis, and power and effect size calculations. Analysis focused on the pretest and posttest differences between the intervention and control groups.	A significant increase in the knowledge and practices of nursing students who received web-based pediatric palliative care education compared to the control group (*p* < 0.05). The intervention group showed statistically significant improvements in their total knowledge scores and self-reported palliative care practices, particularly in subscales related to care during the death stage, patient and family-centered care, pain management, and communication (*p* < 0.05). The web-based education program explained 9.6% of the increase in knowledge level and 36% of the increase in self-reported palliative care practices.	The study did not use random sampling, limiting the generalizability of the findings. The research was conducted at a single university, potentially affecting the broader applicability of the results. The study did not evaluate the long-term retention of the knowledge and skills acquired through the web-based program.
Conner er al. [30]	2014	USA	To evaluate the impact of an online death and dying course on nursing students’ attitudes and feelings about caring for patients at the end of life.	Quantitative quasi-experimental study	123 baccalaureate nursing students (58 in the intervention group and 65 in the control group).	A 16-week online elective course on death and dying, designed to familiarize students with basic end-of-life (EOL). The course included asynchronous assignments, discussions, reflective activities.	Frommelt Attitude Toward Care of the Dying (FATCOD) scale. Death Attitudes Profile-Revised (DAP-R). Data Analysis: Descriptive statistics. Paired and independent *t*-tests. Covariance analysis. Regression analysis.	The online death and dying course significantly improved nursing students’ attitudes towards caring for the dying and acceptance of death. Post-intervention Death Attitudes Profile-Revised scores on the death avoidance and approach acceptance subscales predicted post-intervention FATCOD scores.	Lack of control over the content discussed in the control group. Use of a convenience sample that may limit generalizability. The small number of prelicensure students made meaningful comparisons difficult
Ellman et al. [38]	2012	USA	To create, implement, and evaluate an interprofessional educational program that combines online learning with live interactive simulation to teach spiritual, cultural, and interprofessional aspects of palliative care to students in medicine, nursing, chaplaincy, and social work.	Mixed Methods	Medical, nursing, chaplaincy, and social work students, with 217 completing the online component and 309 submitting post-workshop evaluations.	The program had two components: Online Learning Module: a case-based module that presented a clinical case of a patient with end-stage metastatic breast cancer. Interactive Simulation Workshop: a live 90 min workshop using small group discussions and role-playing scenarios to promote interprofessional collaboration.	Content Analysis: free-text responses to reflection questions during the online module. Post-Workshop Questionnaire: evaluating students’ perceptions of program quality, educational objectives, and usefulness for future professional work. Data analysis: Quantitative analysis: descriptive statistics for Likert-scale items; nonparametric tests (Kruskal–Wallis and Mann–Whitney U tests) to compare scores among different professional groups. Qualitative Analysis: content analysis of free-text responses and open-ended comments from the post-workshop questionnaire.	The program met its educational objectives, with mean responses for all groups exceeding 4 on a 5-point scale. Students valued the program’s ability to enhance understanding of the spiritual and cultural aspects of palliative care and interprofessional collaboration. Medical students rated some aspects of the program lower than nursing and divinity students, possibly due to differences in expectations or less familiarity with interprofessional education. Qualitative data indicated that students appreciated the diversity of professional perspectives and the opportunity for collaborative learning.	The evaluation relied on self-reported measures without assessing higher-level learning outcomes or behavior change. Uneven representation of different professional groups limited generalizability. The study was conducted in a single institution, which may affect its transferability to other educational settings.
Kasar [18]	2023	Turkey	To examine the palliative care practices and self-efficacy status of nursing students taking an online palliative care course.	Quantitative quasi-experimental study	46 nursing students enrolled in the spring term of the 2020–2021 academic year.	An online elective palliative care course conducted over 14 weeks, covering topics such as the concept of palliative care, symptom management, and end-of-life care.	Student Introduction Form: to capture demographic data and perceptions of palliative care. Palliative Care Self-Reported Practices Scale (PCPS): to assess how students implement palliative care recommendations. General Self-Efficacy Scale: to measure the students’ self-efficacy. Data analyses: Descriptive statistics for individual characteristics. Wilcoxon signed-ranks test for PCPS data (not normally distributed). Paired sample *t*-test for General Self-Efficacy Scale (normally distributed). Cronbach’s alpha coefficients were calculated for internal consistency	The online palliative care course significantly improved the students’ self-efficacy and palliative care practices. The mean score on the General Self-Efficacy Scale increased from 63.41 before the course to 68.60 after the course, showing a statistically significant improvement (*p* < 0.05). The mean score on the Palliative Care Self-Reported Practices Scale increased from 69.43 to 81.19, indicating a significant improvement in palliative care practices (*p* < 0.05). A strong positive correlation was found between palliative care practices and self-efficacy after the course (r = 0.402, *p* < 0.05).	The study was conducted in a single institution with a small sample size, limiting the generalizability of the findings. The lack of a control group limits the ability to compare the effects of the intervention to standard or alternative teaching methods.
Mazanec et al. [31]	2019	USA	To develop and implement an innovative online nursing curriculum that prepares associate degree nursing students with essential primary palliative care skills.	Quantitative non-experimental studies	More than 12,000 associate degree nursing students across the United States.	An online e-learning curriculum called ELNEC-Undergraduate, consisting of six one-hour modules.	Student evaluations: Nine questions at the end of each module, using a Likert scale to measure the ease of use, relevance, and impact on clinical practice. Faculty survey: Feedback collected from faculty involved in using the curriculum. Data analysis: Descriptive statistics to analyze student and faculty evaluations. Qualitative insights from open-ended responses to understand the impact on clinical practice and teaching experience	The online curriculum was positively received by both students and faculty, with over 98% of student responses indicating that the modules were relevant and helpful. Faculty appreciated the flexibility and accessibility of the online format, particularly in rural settings where traditional learning methods are less feasible. The curriculum enabled students to gain confidence and competence in primary palliative care skills without requiring extensive modifications to existing syllabi or courses.	Limited generalizability due to the use of a convenience sample. Faculty concerns about time constraints and the cost of the curriculum were noted, though efforts are being made to address these issues.
Price et al. [8]	2015	USA	To investigate the impact of using digital stories to promote a deeper understanding of palliative care concepts among nursing students.	Mixed Methods	134 fourth-year nursing students	Students created 5 min digital stories using VoiceThread technology, synthesizing and applying knowledge about palliative care. The project involved the creation, sharing, and peer feedback of digital stories.	Pre- and post-intervention surveys assessing student perceptions of palliative care competencies and their engagement in the course. Focus group discussions for qualitative feedback on the digital storytelling process and its impact. Data analysis: Descriptive statistics to analyze survey data. Thematic content analysis of focus group transcripts using a grounded theory approach to identify key themes.	Digital storytelling enhanced students’ understanding of the personal and complex nature of palliative care. Students found the digital stories emotionally powerful and effective in increasing their depth of understanding of palliative and end-of-life care concepts. The use of digital storytelling encouraged creativity, promoted affective learning, and helped students engage with complex topics in a personal and meaningful way. Peer feedback and sharing were valued by students, although ranking peer stories was less favored.	The study’s generalizability is limited by the use of a small, convenience sample from a single university. Lack of control over contextual and confounding variables, such as previous experience with palliative care or technology. Possible researcher bias in focus group responses, although efforts were made to minimize this by using a researcher unfamiliar with the students.
Wittenberg et al. [37]	2018	USA	To assess the impact of an online communication training module, developed for undergraduate nursing students, on attitudes, knowledge, and behaviors regarding communication with family caregivers of cancer patients.	Quantitative quasi-experimental study	Undergraduate nursing students, totaling 128 participants (76 from the University of Memphis and 52 from California State University, Los Angeles).	An online educational module derived from the COM-FORT™ SM communication curriculum, specifically tailored to ad-dress communication with family caregivers of cancer patients.	Pre- and Post-Module Surveys: 10-item surveys measuring attitudes, knowledge, and behavior towards communication with caregivers. Open-Ended Questions: included within the module to capture qualitative data on communication knowledge and behavior. Data analysis: Paired Samples *t*-test: assessed pre- and post-module differences in attitudes, knowledge, and behavior. Qualitative Coding: open-ended responses were coded.	A significant increase in students’ communication knowledge, attitudes, and behaviors was observed post-module (*p* < 0.001). The module effectively improved students’ ability to engage with different caregiver types, particularly the Carrier and Manager types. First and fourth-year nursing students showed the greatest gains in attitude, knowledge, and behavior changes. Students demonstrated mastery of communication skills in response to case study scenarios, with 40–56% achieving a mastery level of 2 or higher.	The study lacked a formal course evaluation, limiting the assessment of the module’s effectiveness. Implementation differences between the two nursing programs make comparison challenging and affect reliability. Generalizability is limited due to content variations in nurse communication and cancer care across different programs.
Lewis-Pierre et al. [34]	2019	USA	To explore the impact of two educational approaches online module only and online module plus simulation on nursing students’ attitudes towards caring for patients at the end of life (EOL).	Mixed Methods	Accelerated BSN students in their second semester, totaling 136 participants (71 completed the online module only; 65 completed the module plus simulation).	Online Module: the EOL module covered cultural and religious implications, common fears and concerns of dying patients, signs and symptoms of pain, strategies to manage distressing symptoms, communication techniques, roles of interdisciplinary team members, and the nurse’s role in EOL care. Simulation: A two-part hybrid simulation scenario involving a high-fidelity manikin and a standardized patient (SP).	Frommelt Attitude Toward Care of the Dying Scale version-B (FATCOD-B): Open-ended qualitative questions to explore deeper insights into the students’ experiences and attitudes. Data analysis: Quantitative Analysis: mixed analysis of variance (ANOVA). Qualitative Analysis: thematic analysis was conducted to interpret the open-ended responses.	Both groups showed significant improvement in attitudes towards care of the dying, as indicated by the FATCOD-B scores (*p* = 0.007). The simulation experience was reported to have a more significant impact on students’ attitudes compared to the online module alone, with simulation participants expressing greater confidence and comfort in EOL care. Qualitative data indicated that participants valued the experiential learning provided by the simulation, which was seen as more impactful in preparing them for future practice.	The study did not explore the long-term retention of changes in attitudes, which could differ based on the type of educational intervention. Limited generalizability due to the use of a convenience sample from a single academic institution. Lack of comparison with traditional classroom-based EOL education.
Zhang et al. [32]	2024	China	To develop a virtual clinical simulation education system and assess its impact on enhancing nursing students’ knowledge, ability, and attitudes toward palliative care.	Mixed Methods	76 Third year nursing students.	A virtual clinical simulation education system designed using the Uni-ty3D game engine and accessible through an online learning platform. The system included three modules: Basic Knowledge Module: Practice Theater Module: Test & Quiz Module:	Pre- and Post-Tests: assessed knowledge, ability, and attitudes using three questionnaires: Nurses’ Palliative Care Knowledge Scale Undergraduate Nursing Students’ Palliative Care Ability Assessment Questionnaire. Chinese version of the Frommelt Attitudes Toward Care of the Dying Scale (FATCOD-B) Focus Group Interviews: Conducted to gather qualitative insights on students’ perceptions and experiences. Data analysis: Quantitative Analysis: Paired *t*-tests to compare pre-test and post-test scores for knowledge, ability, and attitudes. Qualitative Analysis: Thematic analysis of focus group interviews to identify key themes regarding the value, usability, and areas for improvement of the virtual clinical simulation.	Significant improvements were observed in students’ palliative care knowledge, ability, and attitudes post-intervention (*p* < 0.001). Students positively evaluated the virtual clinical simulation’s usefulness and usability, citing its value in enhancing knowledge, communication skills, and clinical decision-making abilities. Four key themes emerged from the focus groups: the educational value of the system, its role as a supplement to clinical practice, the enjoyment and accessibility of learning, and the technical challenges encountered.	Limited by a small sample size and the use of a single-group pre-test/post-test design. The system’s lack of realism in simulating non-verbal aspects such as facial expressions and emotional reactions reduced immersion. The study did not employ a more rigorous experimental design, such as a randomized controlled trial, to evaluate the effectiveness of the virtual simulation comprehensively.
Shrestha et al. [33]	2024	Canada	To examine nursing students’ perceptions of a palliative care e-learning module and determine its acceptability.	Cross-sectional survey	Third-year undergraduate nursing students, with 195 respondents out of 249 (78.3% response rate).	A 90 min asynchronous e-learning module covering palliative care, hospice, and medical assistance in dying (MAiD), including videos, evolving case study, and knowledge checks.	Feedback survey with four 5-point Likert items + one open-ended question. Descriptive statistics (mean, SD, frequency). Content analysis for qualitative responses.	Students reported enhanced knowledge, confidence, and positive perceptions toward working with palliative patients. Most found the module enjoyable and well-organized; suggested more content on MAiD and communication.	Cross-sectional design limits causality. Conducted at a single university in Western Canada; limited generalizability. Voluntary participation may introduce bias.
Jeon et al. [35]	2024	South Korea	To evaluate whether a technology-based interactive simulation improves nursing students’ communication skills when handling end-of-life care with grieving caregivers.	Randomized Controlled Trial	Third- and fourth-year undergraduate nursing students, with 80 students exposed to the end-of-life scenario (approximately 40 interventions; 40 control).	End-of-Life Case Only: Simulation involving a 70-year-old patient with terminal colon cancer who dies in hospital. Students observed and practiced communication with a grieving caregiver and coordination with senior nurses for post-death procedures.	Self-reported scales and rater-assessed communication performance—measured pre, post, and 4-week follow-up. Statistical methods included *t*-tests and generalized estimating equation (GEE) models.	Students exposed to the EOL simulation showed significant improvement in compassion and therapeutic communication toward grieving caregivers compared to control. Confidence in handling death-related conversations increased.	EOL simulation was only one of two scenarios in the intervention, so its isolated effect cannot be fully separated from the general program. Conducted in a single cultural context (Korea)—limits generalizability.
Flood [36]	2024	USA	To explore whether embodying a terminally ill patient at end-of-life using immersive VR changes students’ confidence, emotional responses, and perceptions toward hospice and EOL communication.	Quasi-experimental pre/post	Bachelor of Science in Nursing students, with 32 students completing the end-of-life simulation.	EOL VR Scenario Only: Students embodied “Clay”, a 66-year-old hospice patient with stage IV lung cancer, progressing through final months → final weeks → final minutes → im-mediate post-death care by staff. VR delivered via Oculus Rift (Embodied Labs).	Pre/post confidence scales (1–10), Likert items on hospice perception, checklist of emotions be-fore vs. after simulation, open-ended qualitative responses. Wilcox-on signed-rank & McNemar’s tests.	Confidence in providing care in last months, weeks, and final minutes of life increased significantly. Positive perceptions of hospice improved. Negative emotions like fear and sadness decreased; positive emotions like peacefulness, empowerment increased. 93% reported it would change how they discuss end-of-life with patients.	Small convenience sample from one university. No control group for comparison. No debriefing provided post-simulation, which may limit reflective learning. Some emotional distress or cybersickness reported.

## Data Availability

No new data was created. Data sharing is not applicable to this article.

## Supplementary Materials

The following supporting information can be downloaded at: https://www.mdpi.com/article/10.3390/nursrep16010016/s1. Supplementary File S1—PRISMA Checklist. Supplementary File S2—CINAHL Search String. Supplementary File S3—Quality Appraisal Results.

## Public Involvement Statement

No public involvement in any aspect of this research.

## Guidelines and Standards Statement

This manuscript was drafted against the PRISMA [19] reporting guideline for systematic reviews.

## Abbreviations

The following abbreviations are used in this manuscript:

EAPC	European Association for Palliative Care
IAHPC	International Association for Hospice and Palliative Care
WHO	World Health Organization
PRISMA	Preferred Reporting Items for Systematic Reviews and Meta-Analyses
OSF	Open Science Framework
MeSH	Medical Subject Headings
MMAT	Mixed Methods Appraisal Tool
MOOC	Massive Open Online Course
VR	Virtual Reality
SRPCP	Scale of Self-Reported Palliative Care Practices
FATCOD	Frommelt Attitude Toward Care of the Dying
DAP-R	Death Attitudes Profile–Revised
PCPS	Palliative Care Self-Reported Practices Scale
EOL	End of Life
MAiD	Medical Assistance in Dying
PC	Palliative Care
