# Supplementary material for: Digital Interventions for Palliative Care Education for Nursing Students: A Systematic Review
Source: Nurs Rep. 2026 Jan 7;16(1):16. doi: 10.3390/nursrep16010016 (PMC12844837; doi:10.3390/nursrep16010016)
Supplement: Supplementary file 1 [file nursrep-16-00016-s001.zip › Supplementary File 2 CINAHL Search.pdf]

Supplementary File 2:
CINAHL Search Strategy:

(("digital learning" OR "e-learning" OR "online education" OR "web-based learning" OR "virtual simulation" OR "serious games") AND ("nursing student*" OR "pre-registration nursing") AND ("palliative care" OR "end-of-life care" OR "hospice care")).
