# Supplementary material for: Digital Interventions for Palliative Care Education for Nursing Students: A Systematic Review
Source: Nurs Rep. 2026 Jan 7;16(1):16. doi: 10.3390/nursrep16010016 (PMC12844837; doi:10.3390/nursrep16010016)
Supplement: Supplementary file 1 [file nursrep-16-00016-s001.zip › Supplementary File 3- MMAT.pdf]

Supplementary File 3:

Each study was evaluated according to the appropriate MMAT checklist criteria, with appraisal criteria applied according to study design, including appropriateness of outcome measures, adequacy of data collection and analysis, consideration of confounders, and coherence between qualitative and quantitative components if applicable. This methodology ensures that the conclusions derived from these studies are founded on robust, high-quality evidence, which is essential for synthesising findings related to digital interventions in palliative care education for pre-registered nursing students. All 12 studies were considered for inclusion in the review, none of the studies were excluded due to being too weak. While some studies are moderate quality, all were deemed sufficiently robust to be included in the review. The studies ranged from moderate to high quality levels, but none were excluded for being of insufficient quality.

MMAT Table

Author and Year	Study Type	MMAT Score	Quality of The Study
**Akdeniz and Bektas, (2020)**	Quantitative quasi-experimental study	Moderate	Moderate quality study
**Conner er al., (2014)**	Quantitative quasi-experimental study	Moderate	Moderate quality study
**Ellman et al., (2012)**	Mixed Methods	High	High quality study
**Kasar (2023)**	Quantitative quasi-experimental study	High	High quality study
**Mazanec et al., (2019)**	Quantitative non-experimental study	High	High quality study
**Price et al., (2015)**	Mixed Methods	High	High quality study
**Wittenberg et al., (2018)**	Quantitative quasi-experimental study	Moderate	Moderate quality study

**Lewis-Pierre et al., (2019)**	Mixed Methods	High	High quality study
**Zhang et al., (2024)**	Mixed Methods	High	High quality study
**Shrestha et al. (2024)**	Cross-sectional survey	Moderate	Moderate quality study
**Jeon et al., 2024**	Randomized Controlled Trial	High	High quality study
**Flood, L.S., 2024**	Quantitative, Quasi-experimental pre/post	Moderate	Moderate quality study

Justification:

Akdeniz & Bektas (2020) – Quantitative quasi-experimental

The study addressed a clearly defined research question and employed appropriate outcome measures and statistical analyses. Limitations related to non-random sampling, single-site design, and lack of long-term follow-up reduced robustness. Overall, the study was rated as **moderate quality**.

Conner et al. (2014) – Quantitative quasi-experimental

This study met MMAT criteria relating to clarity of research questions, appropriateness of outcome measures, and statistical analysis. However, the absence of randomisation, reliance on a convenience sample, and limited control over the comparator group reduced internal validity, confounding variables were not fully addressed. Overall, the study was rated as **moderate quality**.

Ellman et al. (2012) – Mixed methods

The study showed strong coherence between aims, intervention, and outcomes, with appropriate use of both quantitative and qualitative data. Integration was clearly articulated. Limitations were primarily contextual rather than methodological. The study was rated as **high quality**.

Kasar (2023) – Quantitative quasi-experimental

Although the study employed a quasi-experimental design without a control group, the study met all MMAT criteria for quantitative non-randomised studies, including appropriate outcome measurement, adequate handling of confounders within the study design, and robust statistical analysis. Therefore, it was rated as **high quality**.

Mazanec et al. (2019) – Quantitative non-experimental

This large-scale evaluation clearly defined its aims, intervention, and outcome measures. Data collection and analysis were appropriate for the study design, although the absence of a comparator and reliance on self-reported evaluations limited causal inference. Given the consistency, scale, and transparency of reporting, the study was rated as **high quality**.

Price et al. (2015) – Mixed methods

The study demonstrated strong alignment between research questions, data collection, and analysis. Quantitative and qualitative components were well integrated, and the qualitative analysis was clearly described. Limitations related mainly to sampling and contextual factors rather than methodological flaws. This study met most MMAT criteria and was rated as **high quality**.

Wittenberg et al. (2018) – Quantitative quasi-experimental

The study demonstrated clear aims and appropriate analytical methods. However, implementation variability across sites and the absence of a formal course evaluation weakened methodological consistency. Confounding factors were not fully controlled. The study was rated as **moderate quality**.

Lewis-Pierre et al. (2019) – Mixed methods

This study demonstrated methodological rigour across both quantitative and qualitative components. Integration of findings was explicit and meaningful, enhancing

interpretability. While generalisability was limited by sampling, MMAT criteria were largely satisfied, supporting a **high-quality** appraisal.

Zhang et al. (2024) – Mixed methods

This study met MMAT criteria across qualitative, quantitative, and integration domains. The mixed-methods design was well justified, data sources were coherent, and findings were clearly integrated. Despite limitations related to sample size and design, methodological rigour supported a **high-quality** rating.

Shrestha et al. (2024) – Quantitative descriptive (cross-sectional)

The study met MMAT criteria for descriptive designs, including clarity of measures and appropriateness of analysis. However, the cross-sectional design, voluntary participation, and reliance on self-report limited causal interpretation. The study was therefore rated as **moderate quality**.

Jeon et al. (2024) – Quantitative randomised controlled trial

This study met MMAT criteria relating to randomisation, outcome measurement, and statistical analysis. While blinding was not fully described and cultural specificity may limit transferability, the overall methodological quality was strong. The study was rated as **high quality**.

Flood (2024) – Quantitative quasi-experimental

The study employed appropriate measures and analysis for its design; however, the absence of a control group, small sample size, and lack of structured debriefing reduced methodological strength. Confounders were not fully addressed. The study was rated as **moderate quality**.
